# Edaravone Protected Human Brain Microvascular Endothelial Cells from Methylglyoxal-Induced Injury by Inhibiting AGEs/RAGE/Oxidative Stress

**DOI:** 10.1371/journal.pone.0076025

**Published:** 2013-09-30

**Authors:** Wenlu Li, Hongjiao Xu, Yangmin Hu, Ping He, Zhenzhen Ni, Huimin Xu, Zhongmiao Zhang, Haibin Dai

**Affiliations:** 1 Department of Pharmacy, Second Affiliated Hospital, Zhejiang University School of Medicine, Hangzhou, Zhejiang, China; 2 Department of Anesthesiology, Second Affiliated Hospital, Zhejiang University School of Medicine, Hangzhou, Zhejiang, China; 3 Cancer Institute, Zhejiang University, Hangzhou, Zhejiang, China; Case Western Reserve University, United States of America

## Abstract

Subjects with diabetes experience an increased risk of cerebrovascular disease and stroke compared with nondiabetic age-matched individuals. Increased formation of reactive physiological dicarbonyl compound methylglyoxal (MGO) seems to be implicated in the development of diabetic vascular complication due to its protein glycation and oxidative stress effect. Edaravone, a novel radical scavenger, has been reported to display the advantageous effects on ischemic stroke both in animals and clinical trials; however, little is known about whether edaravone has protective effects on diabetic cerebrovascular injury. Using cultured human brain microvascular endothelial cells (HBMEC), protective effects of edaravone on MGO and MGO enhancing oxygen-glucose deprivation (OGD) induced injury were investigated. Cell injury was measured by 3-(4,5-Dimethylthiazol-2-yl)-2,5-diphenyltetrazolium bromide (MTT) formation, cell account, lactate dehydrogenase (LDH) release and Rhodamine 123 staining. Advanced glycation end-products (AGEs) formation and receptor for advanced glycation end-products (RAGE) expression were measured by western blotting. Cellular oxidative stress was measured by reactive oxygen species (ROS) release. Treatment of MGO for 24 h significantly induced HBMEC injury, which was inhibited by pretreatment of edaravone from 10–100 µmol/l. What’s more, treatment of MGO enhanced AGEs accumulation, RAGE expression and ROS release in the cultured HBMEC, which were inhibited by 100 µmol/l edaravone. Finally, treatment of MGO for 24 h and then followed by 3 h OGD insult significantly enhanced cell injury when compared with OGD insult only, which was also protected by 100 µmol/l edaravone. Thus, edaravone protected HBMEC from MGO and MGO enhancing OGD-induced injury by inhibiting AGEs/RAGE/oxidative stress.

## Introduction

Cerebrovascular disease remains a leading cause of morbidity and mortality in subjects with diabetes distinguished by poor glycemic control and impaired glucose tolerance [1]. It has been documented that hyperglycemia exacerbates ischemic stroke, which is associated with augmentation in the size of the infarct and vasodegenerative change [2], [3]. Although many factors contribute to cerebrovascular dysfunction in diabetes, it is now widely accepted that methylglyoxal (MGO) plays a critical role in the progression of diabetic vascular complications. In hyperglycemic conditions, levels of precursors of triose phosphate, such as glucose or fructose, are increased. After nonenzymatic fragmentation, high serum levels of MGO were observed in patients with either type 1 or type 2 diabetes [4]. MGO as one of the most reactive dicarbonyls, is considered to be an important glycating agent to consider for glycation damage to the mitochondrial proteome [5]. Moreover, the cytotoxicity of MGO is mediated by the modification of deoxyribonucleic acid (DNA) and activation of apoptosis [6].

Vascular disorders will induce several biochemical and cellular reactions such as inflammatory response, increased reactive oxygen species (ROS) production, impairment of blood brain barrier and calcium overload [7]. Edaravone, the first clinical drug of neuroprotection for ischemic stroke patients in the world, is used for the purpose of aiding neurological recovery following acute brain ischemia and subsequent cerebral infarction [8], [9]. In a recent study, edaravone has been proved to modulate endothelial barrier properties via the activation of S1P1 and a downstream signaling pathway [10]. These findings provide new insights for edaravone as an effective therapeutic agent for diseases with systemic vascular endothelial disorders such as diabetes stroke.

The present study was aimed to demonstrate the protective effect of edaravone on MGO-induced injury in the cultured human brain microvascular endothelial cells (HBMEC) and accompanied by identifying the possible mechanism which is responsible for the protection. What’s more, the protective effect of edaravone was also investigated in MGO enhancing oxygen-glucose deprivation (OGD) induced injury. Data derived from the present study raise the possibility that edaravone may be a new strategy to prevent or improve vascular complications associated with diabetes stroke.

## Materials and Methods

### Materials

HBMEC (Stins et al. 2001) were cultured in RPMI 1640 and characterized for brain endothelial phenotypes as previously described [11], [12]. RPMI 1640, fetal bovine serum, Nu-Serum, MEM non-essential amino acids, sodium pyruvate were purchased from Gibco (Grand Island, USA). Edaravone (purity ≥98%), methylglyoxal (MGO, 40% w/v) were purchased from Yuanye Biotech (Shanghai, China). 3-(4,5-Dimethylthiazol-2-yl)-2,5-diphenyltetrazolium bromide (MTT), aminoguanidine were purchased from Sigma (St. Louis, MO, USA). Lactate dehydrogenase (LDH) assay kit and ROS assay kit were purchased from Nanjing Institute of Jianchen Biological Engineering (Nanjing, China). Rhodamine 123 using the apoptosis detection kit was purchased from KeyGen Biotech (Nanjing, China). Mouse anti-β-actin monoclonal antibody was purchased from Abmart (Shanghai, China). Goat anti-advanced glycation end-products (AGEs) polyclonal antibody was purchased from Millipore (Billerica, MA, USA). Rabbit anti-receptor of advanced glycation end-products (RAGE) polyclonal antibody was purchased from Abcam (Cambridge, MA, USA). All the secondary antibodies were purchased from Multi Sciences (Hangzhou, China).

### HBMEC Treatments

HBMEC were treated with 1 mmol/l aminoguanidine or the indicated concentrations (from 10 to 100 µmol/l) of edaravone 20 min prior to MGO (2 mmol/l) treatment, and then cells were incubated under OGD condition for another 3 h. Aminoguanidine, a nucleophilic hydrazine compound, was adopted as a positive control [13].

### Cell Viability and Cell Counting Measurements

Cell viability was determined by the MTT assay. Equal numbers of the cultured HBMEC were exposed to various treatments, and cells were incubated with an MTT solution (50 µl, 5 mg/ml) at 37°C for 4 h. Next, cells were placed in wells of a microtiter plate and scanned to visualize the color development. Cell survival rates were expressed as percentages of the value of normal cells. Cell numbers were counted by trypan blue dye exclusion analysis. Briefly, culture medium was replaced by phosphate buffer saline (PBS)/0.4% trypan blue, which stains nonviable cells. The data represented 6–12 separate wells assayed per data point, with about 500–1,000 cells counted per well.

### BSA- MGO Assay

BSA- MGO assay was adopted from previous report with some modification [14], which was used for investigation of inhibitors on the middle stage of the glycation of protein [15]. BSA (50 mg/ml) was incubated with MGO (100 mmol/l) under sterile, dark conditions in 0.1 mmol/l phosphate buffer (pH 7.4) at 37°C for 24 h in the presence or absence of various concentrations of the compounds. In certain experiments, the indicated edaravone was added to the model system in the concentration range of 0.01–10 mmol/l. Fluorescence of the samples was measured at the excitation and emission maxima of 330 nm and 410 nm, respectively. AG (50 mmol/l) was used as a positive control. The effects of MGO modification on the cross-linking and aggregation of BSA were investigated by sodium dodecyl sulfate polyacrylamide gel electronphoresis (SDS-PAGE) using a 4% stacking and 12% separating gel. Protein map was visualized by Coomassie blue stain.

### Cell Death and Apoptosis

The amount of LDH released by cells was determined by an LDH assay kit according to manufacture’s protocol. Cell apoptosis was measured by Rhodamine 123 using apoptosis detection kit according to the manufacture’s instructions. Briefly, the cell suspension was incubated at room temperature with Rhodamine 123 for 10 min in the dark. For each sample, at least 1×10^4^ cells were analysed using a fluorescence activated cell sorter (FACS) Calibur flow cytometer.

### Western Blotting

Fifty-microgram protein samples were loaded on 12% SDS-PAGE gels and blotted onto polyvinylidene fluoride (PVDF) membranes, which were probed with primary antibodies against RAGE (1∶2000), against AGEs (1∶1000) and β-actin (1∶2000) at 4°C overnight. Horseradish peroxidase-conjugated anti-mouse, anti-rabbit and anti-goat secondary antibodies were then used at room temperature for 1 h; signals were detected by chemiluminescence.

### ROS Release

The amount of ROS released by cells was determined by a ROS assay kit according to manufacture’s protocol. Briefly, the cultured HBMEC were incubated with 2,7-dichlorofluorescein-diacetate (DCFH-DA) (10 µmol/l) at 37°C for 6 h, after washing by PBS for 3 times, cells were placed in wells of a microtiter plateand scanned to visualize the color development. ROS release was expressed as folds increase of the value of normal cells.

### Oxygen-glucose Deprivation Exposure

To mimic ischemic-like conditions *in vitro*, cell cultures were exposed to permanent glucose deprivation and hypoxia for 3 h. In the oxygen and glucose-deprivation phase, the cells were washed with RPMI 1640 and changed to glucose-free medium, after which the cultures were placed in an airtight experimental hypoxia chamber (Billups-Rothen-berg, San Diego, CA, USA) containing a gas mixture composed of 95% N_2_/5% CO_2_.

### Statistical Analysis

All data were expressed as mean ± SD. Statistical analysis was performed by one-way ANOVA followed by the Newman-Keuls test for multiple comparison tests (using SPSS 16.0 software; SPSS Inc., Chicago, USA). Statistical significance was set at P<0.05.

## Results

### MGO Concentration- and Time- dependently Induced Cell Injury in the Cultured HBMEC

**Figure 1:**
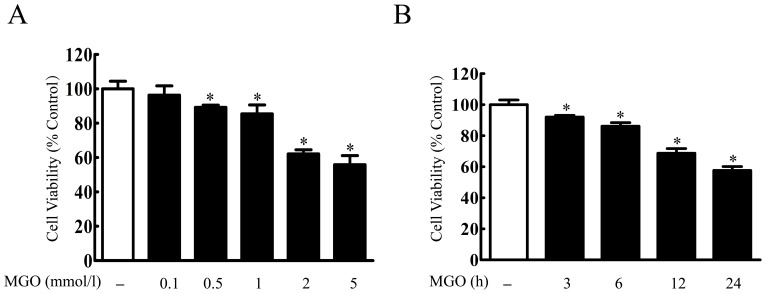
MGO concentration-dependently induced cell injury in the cultured HBMEC. (A) HBMEC were incubated with different concentration of MGO (100 µmol/l, 500 µmol/l, 1 mmol/l, 2 mmol/l and 5 mmol/l) for 24 h. (B) HBMEC were incubated with MGO (2 mmol/l) for 3, 6, 12, 24 h. Cell viability was determined by MTT assay. ^*^p<0.05 versus control group. n = 3 repeats.

First, effect of MGO on the cultured HBMEC was investigated. Treatment of MGO for 24 h decreased cell viability in a concentration-dependent manner from 100 µmol/l to 5 mmol/l. At 5 mmol/l, the cell viability was only 55.83±5.33% (Fig. 1A). We then determined the temporal changes in endothelial cell death 3–24 h after 2 mmol/l MGO exposure. The viability of cells subjected to MGO showed a time-dependent decrease in viability when exposed to MGO for 3 h (92.06±1.12%), 6 h (86.19±2.27%), 12 h (68.79±2.99%), and 24 h (57.67±2.44%) compared with control cells (Fig. 1B).

### Edaravone Protected MGO-induced Injury in the Cultured HBMEC

**Figure 2:**
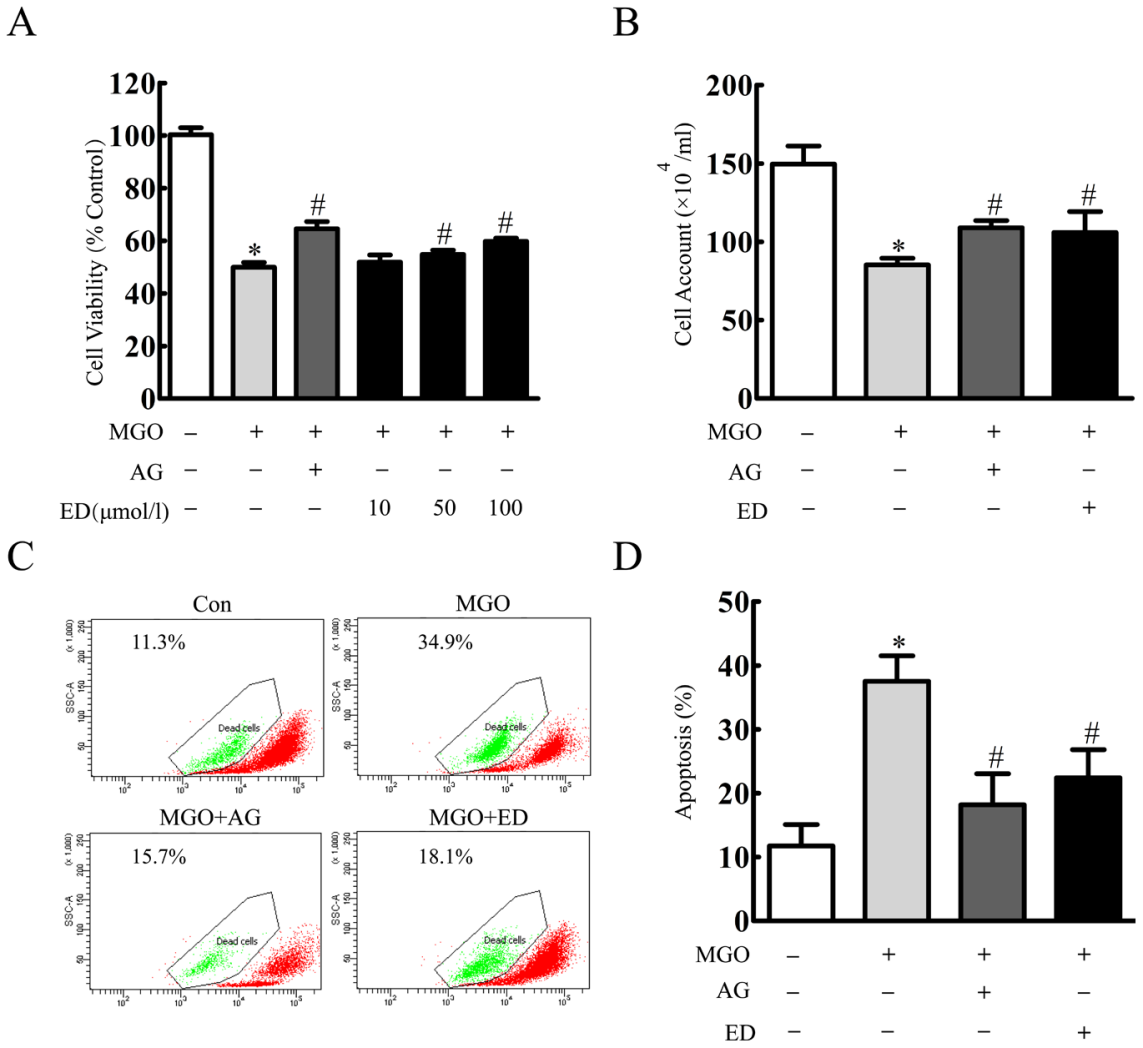
Edaravone protected MGO-induced cell injury in the cultured HBMEC. (A) HBMEC were incubated with edaravone (10 µmol/l, 50 µmol/l, 100 µmol/l), aminoguanidine (1 mmol/l) for 20 min before MGO (2 mmol/l) treatment. After 24 h MGO treatment, cell viability was determined by MTT assay. (B) Cell counts were assessed by trypan blue exclusion. (C) HBMEC were incubated with edaravone (100 µmol/l), aminoguanidine (1 mmol/l) 20 min before MGO (2 mmol/l) treatment. After 24 h MGO treatment, cell apoptosis was detected by rhodamine 123 staining and flow cytometer. ^*^p<0.05 versus control group. ^#^p<0.05 versus MGO group. n = 3 repeats. AG represented aminoguanidine. ED represented edaravone.

Incubation of 10–200 µmol/l edaravone for 24 h did not affect cell viability (data not shown). HBMEC were pretreated with 1 mmol/l aminoguanidineor 10–100 µmol/l edaravone for 20 min, and then incubated with 2 mmol/l of MGO for 24 h. Cell viability was obviously decreased to 49.94±1.88% in MGO group. In contrast, edaravone mediated high upregulation of cell viability to 54.85±1.63% and 59.77±1.21% at 50 and 100 µmol/l, respectively (Fig. 2A). As measured by cell account, similar protective effects of edaravone were also observed (Fig. 2B). Two mmol/l MGO treatment for 24 h increased cell apoptosis to 37.53±3.97%. When incubation of 1 mmol/l aminoguanidine or 100 µmol/l edaravone 20 min prior to 24 h MGO treatment, cell apoptosis were decreased to 18.20±4.86% and 22.40±4.40%, respectively (Fig. 2C, D).

### Edaravone Inhibited MGO-induced AGE-RAGE Axis and Cellular Oxidative Stress

**Figure 3:**
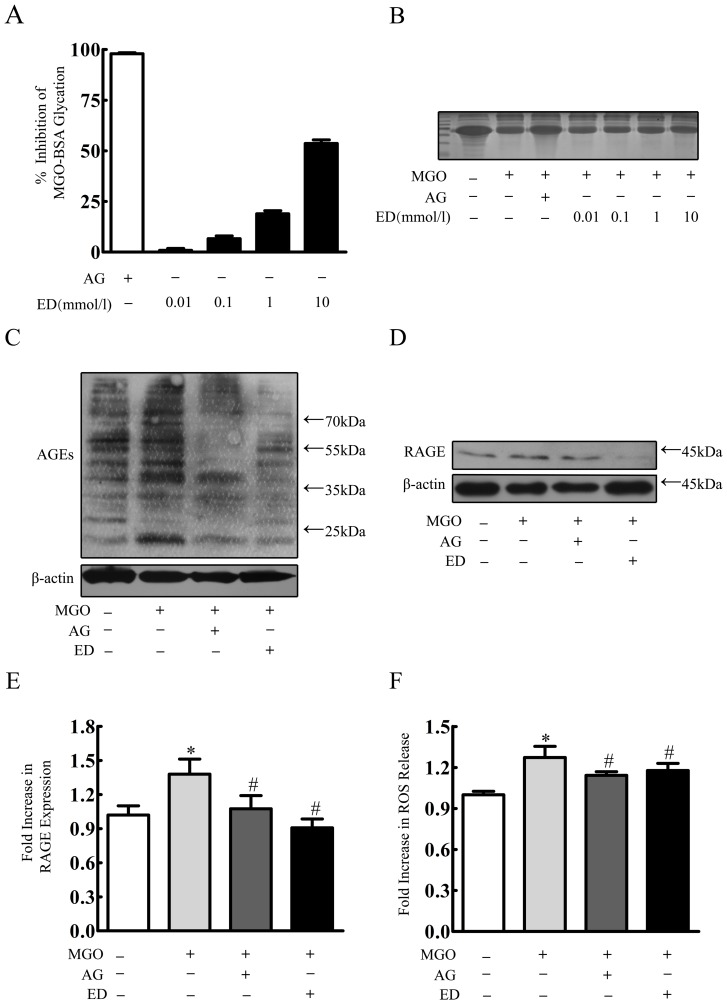
Edaravone inhibited MGO-induced AGE-RAGE axis and cellular oxidative stress. (A) BSA-MGO assay. (B) The modulation of MGO-mediated BSA modification as revealed by SDS-PAGE. Results are means ± S.D. (C, D, E) HBMEC were incubated with edaravone (100 µmol/l), aminoguanidine (1 mmol/l) 20 min before MGO (2 mmol/l) treatment. After 24 h MGO treatment, RAGE, AGEs were determined by Western blotting. (F) HBMEC were incubated with edaravone (100 µmol/l), aminoguanidine (1 mmol/l) 20 min before MGO (2 mmol/l) treatment. After 24 h MGO treatment, cellular oxidative stress was determined by ROS release. ^*^p<0.05 versus control group. ^#^p<0.05 versus MGO group. n = 3 repeats. AG represented aminoguanidine. ED represented edaravone.

As AGEs accumulation was implicated as a key pathogenic process in diabetic complications [16], effects of edaravone on AGEs accumulation and its followed signaling were further investigated. First, we used BSA- MGO assay to determine whether edaravone can inhibit AGEs formation *in vitro*. As shown in Fig. 3A, edaravone concentration dependently decreased BSA glycation as measured by fluorescence. At the concentration of 0.01, 0.1, 1 and 10 mmol/l, edaravone showed significant inhibitory effects by 0.9±0.9%, 6.61±1.38%, 18.92±1.56% and 53.6±1.82%, respectively. Aminoguanidine (50 mmol/l) also exhibited inhibitory effects by 97.9±0.52%. MGO reacts with protein residues such as lysine to produce high molecular weight, cross-linked products [17]. As shown in Fig. 3B, there was a detectable decrease amount of BSA in its usual position when treated with MGO. However, when aminoguanidine or edaravone was added in the incubation mixture, the loss of BSA was inhibited.

Next, we further determined whether edaravone can inhibit AGEs accumulation in the cultured HBMEC. As shown in Fig. 3C, treatment of 2 mmol/l MGO for 24 h significantly increased AGEs accumulations, which was alleviated by pretreatment of 1 mmol/l aminoguanidine or 100 µmol/l edaravone, respectively. A critical property of AGEs is their ability to activate RAGE, a signal transduction receptor of the immunoglobulin superfamily [18]. We also found RAGE expression shared a same trend with AGEs accumulation in MGO-induced cell injury pretreated with edaravone or not (Fig. 3D, E). Due to the fact that RAGE can activate diverse signal transduction cascades and downstream pathways, including generation of ROS, and which plays a vital role in MGO-induced injury in endothelial cells, neuron and macrophage, we then investigated the ROS release among these groups [19]–[22]. After 24 h MGO treatment, ROS release was 1.27±0.08 folds of control group. Pretreatment of aminoguanidine or edaravone significantly decreased the amount of ROS release to 1.14±0.03 and 1.18±0.05 folds of control group, respectively (Fig. 3F).

### Edaravone Protected MGO Enhanced OGD-induced Injury in the Cultured HBMEC

**Figure 4:**
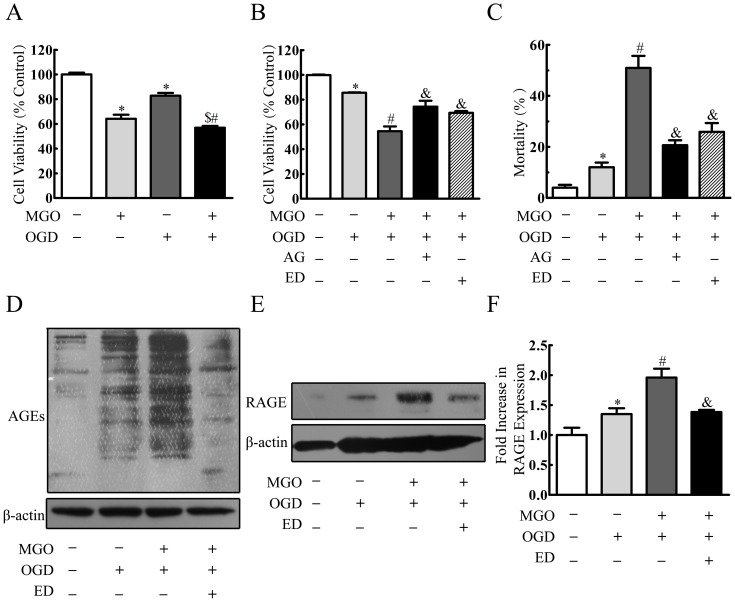
Edaravone protected MGO enhanced OGD-induced injury in the cultured HBMEC. (A) HBMEC were incubated with MGO (2 mmol/l) for 24 h before 3 h OGD. Cell viability was determined by MTT assay. (B) HBMEC were incubated with edaravone (100 µmol/l), aminoguanidine (1 mmol/l) 20 min before MGO (2 mmol/l) treatment. After 24 h MGO treatment, the cultured HBMEC was incubated under OGD condition for another 3 h. Cell viability was determined by MTT assay. (C) Cell mortality was determined by LDH assay. (D, E, F) HBMEC were incubated with edaravone (100 µmol/l) 20 min before MGO (2 mmol/l) treatment. After 24 h MGO treatment, the cultured HBMEC was incubated under OGD condition for another 3 h. RAGE, AGEs were determined by Western blotting. ^$^p<0.05 versus MGO group. *p<0.05 versus control group. ^#^p<0.05 versus OGD group. ^&^p<0.05 versus MGO+OGD group. n = 3 repeats. AG represented aminoguanidine. ED represented edaravone.

A diabetes-enhanced ischemic stroke is associated with reduced formation of capillaries and a reduction in blood flow versus nondiabetic mice [23]. So we further investigated whether MGO can enhance OGD insult in the cultured HBMEC. As shown in Fig. 4A, 3 h OGD insult decreased cell viability to 82.90±2.20%, which was decreased to 56.93±1.46% when pretreated 2 mmol/l MGO for 24 h (Fig. 4A). Edaravone and aminoguanidine protected MGO enhancing OGD induced injury by reversing the cell viability to 69.30±1.50% and 54.51±3.83%, respectively (Fig. 4B). Similar results were also found as measured by LDH release assay (Fig. 4C).

To further verify whether AGEs or RAGE plays an important role on MGO enhancing OGD-induced injury in the cultured HBMEC, accumulation of AGEs and expression of RAGE were investigated. In OGD group, both AGEs accumulation and RAGE expression were increased, and in MGO+OGD group the level of AGEs and RAGE were elevated obviously, whereas AGEs accumulation and RAGE expression significantly decreased by pretreatment of edaravone (Fig. 4D–F).

## Discussion

In the present study, we demonstrated that MGO induced injury was associated with AGEs accumulation, enhancing RAGE expression and ROS release in the cultured HBMEC, which were alleviated by pretreatment of edaravone. Furthermore, MGO enhanced OGD-induced cell injury which was also protected by edaravone.

It is well known that diabetes gain high serum levels of glucose or fructose-precursors of triose phosphate which would augment MGO formation by Maillard reaction [1], [24]. The dicarbonyl compound MGO is involved in a variety of detrimental processes under hyperglycemic conditions [24]. In the present study, we provided evidence that MGO alone could induce the cultured HBMEC damage. MGO increases glycation of mitochondrial proteins which is associated with increased formation of ROS and increased proteome damage by oxidative and nitrosative processes [25]. Edaravone has been reported to display the advantageous effects by protecting against oxidative stress on ischemic stroke both in animals and clinical trials [26], [27]. Notably, the effect of edaravone on diabetic cerebrovascular injury is still unclear. Our present work indicated that MGO-induced injury in the cultured HBMEC could be suppressed by edaravone treatment.

In the development of diabetic complications, MGO reacted on and modified cellular proteins to form cross-links of amino groups, and then generates AGEs [28]. To gain further insight into the mechanism, the temporal changes in AGEs protein levels following MGO treatment were addressed. Our results demonstrated that AGEs accumulation significantly increased after 24 h MGO treatment which was consistent with our previous reports [12], [14]. Intriguingly, edaravone could decrease AGEs accumulation, which was known to impair cellular function by increasing cellular oxidative stress on binding to their specific cell surface receptors, such as RAGE and galectin-3 [22], [29]. RAGE has been linked to several chronic diseases, which are thought to result from vascular damage [29]. Consistent with this finding, we obtained similar results indicating that increase accumulation of AGEs resulted in upregulation of RAGE expression and ROS release [16], [29]. Treatment of cultured HBMEC with edaravone before the course of MGO exposure profoundly inhibited the AGEs accumulation, RAGE expression and damage-induced ROS release, suggesting that edaravone could offer the cultured HBMEC protection against cellular oxidative stress.

It is becoming clear that the integrity of cerebral blood vessels is critical in the pathophysiology of stroke [30]. Accordingly, MGO enhancing OGD-induced injury in the cultured HBMEC in this study seems to be relevant to hyperglycemia exacerbates ischemic stroke *in vivo*. Interestingly, our current study proved that edaravone could also suppress MGO enhancing OGD-induced injury in the cultured HBMEC. Both experimental data and clinical observations displayed the association between MGO enhancing OGD-induced injury and increased endothelial cell damage.

Taken together, it is the first time that our study was designed to systematically investigate the effect of edaravone on inhibition of MGO induced injury *in vitro*. Cells were treated with the MGO, followed with AGEs accumulation and RAGE expression increased. Therefore, edaravone elicited its protective effect via AGEs/RAGE inhibition and this led to further suppress of ROS release. Of interest, the present data showed an augmentation of OGD-induced injury in the cultured HBMEC treated with MGO, which partially decreased by pretreatment with edaravone. Although the identities of pathways may be regulated by edaravone remain to be defined in further study, data from this study showed a novel strategy to diabetic vascular complications and also prevent ischemic stroke associated with diabetes.
